# Enhanced Thermoelectric Properties of a Semiconducting Two-Dimensional Metal–Organic Framework via Iodine Loading

**DOI:** 10.1021/acsami.2c20770

**Published:** 2023-01-23

**Authors:** Maria de Lourdes Gonzalez-Juarez, Mark A. Isaacs, Darren Bradshaw, Iris Nandhakumar

**Affiliations:** † School of Chemistry, University of Southampton, SouthamptonSO17 1BJ, U.K.; ‡ Department of Chemistry, University College London, LondonWC1H 0AJ, U.K.; § HarwellXPS, Research Complex at Harwell, RAL, Harwell Campus, DidcotOX11 0FA, U.K.

**Keywords:** conducting, metal−organic frameworks, dip coating, iodine, thermoelectrics

## Abstract

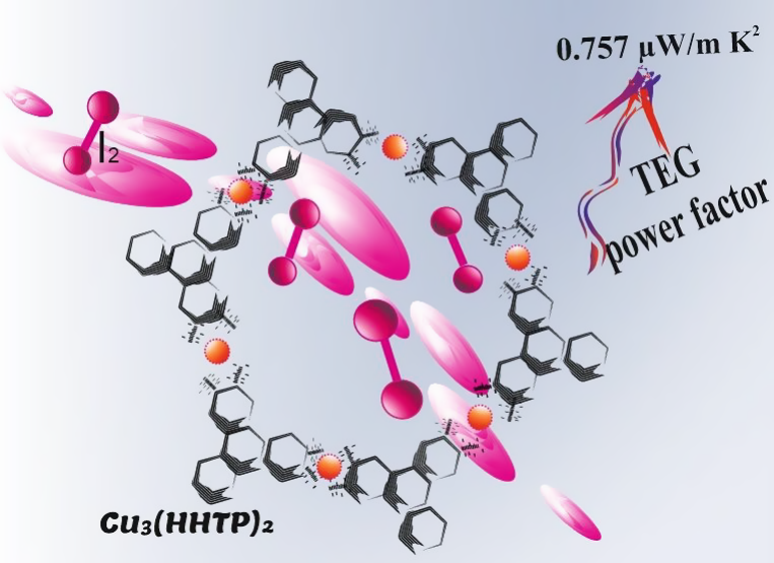

We report the first result of a study in which molecular iodine has been incorporated via incipient wetness impregnation into the two-dimensional semiconducting metal–organic framework (MOF) Cu_3_(2,3,6,7,10,11-hexahydroxytriphenylene)_2_ Cu_3_(HHTP)_2_ to enhance its thermoelectric properties. A power factor of 0.757 μW m^–1^ K^–2^ for this MOF was obtained which demonstrates that this provides an effective route for the preparation of moderate-performance thermoelectric MOFs.

## 1 Introduction

The rise in greenhouse gas emissions and worldwide energy demand requires urgent development of efficient energy conversion technologies that are capable of harvesting energy from natural sources. Thermoelectric generators (TEG) are devices capable of converting heat into electrical energy based on the Seebeck or Peltier effect and are considered to be a promising clean technology to conventional energy sources such as fossil fuels.^1,2^ These devices do not require complex mechanical structures, maintenance is inexpensive, and can significantly reduce the environmental impact of air pollution.^3^ One of the fastest growing areas for thermoelectric technology is the recovery of waste heat in domestic and portable applications. To achieve this goal, it is necessary to find cheaper and facile-to-process nontoxic materials that also perform well at ambient temperatures. The performance of thermoelectric devices is related to the ratio of the electrical energy produced to the thermal energy entering the device, and this is measured by the dimensionless figure of merit *zT* which is given by the following equation:
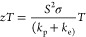


where *S* corresponds to the Seebeck coefficient (V K^–1^), σ is the electrical conductivity (S m^–1^), *k* is the thermal conductivity (W m^–1^ K^–1^) which has two contributions *k*_p_ and *k*_e_, the electronic and phononic contribution, respectively, and *T* is the absolute temperature. In order to achieve a high-performance TEG, a material with high electrical conductivity and low thermal conductivity is desirable, which is the starting point for the design of novel materials for this application.^4,5^

It has been almost a decade since Yaghi et al.^6^ reported the first examples of intrinsically conductive metal–organic frameworks (MOFs) comprising square-planar transition metal ions coordinated to highly conjugated redox active ligands. The effective charge transport observed in these extended two-dimensional (2D) structures is attributed to the excellent orbital conjugation between metal centers and the *ortho*-disubstituted benzene organic units.^7^ The honeycomb layer-stacked 2D structure in these frameworks resembles that observed in graphite. Electrical conductivity measurements conducted on this series of MOFs (in which Ni^2+^, Co^2+^, and Cu^2+^ were employed as metal centers coordinated to 2,3,6,7,10,11-hexahydroxytriphenylene (HHTP) linkers) showed that the Cu analogue possessed the highest conductivity, reaching values of around 0.2 S cm^–1^.

The inherent porosity in MOFs makes them suitable candidates to investigate the modulation of their physical and chemical properties via the incorporation of guest molecules. This process does not disrupt the integrity of the MOFs’ crystal structure. The incorporation of guest molecules within the MOF pores to promote long-range electron delocalization and/or inject free charge carriers (e.g., by oxidizing or reducing the electroactive building units) has become an effective strategy to enhance the electrical properties of MOFs.^8−10^

Different approaches have been employed to introduce guest molecules into MOF species such as co-evaporation of the host framework and guest entity under vacuum,^11^ mixing the host and guest materials in the liquid phase (incipient wetness impregnation) followed by its physical deposition (e.g., dip coating)^12^ or diffusion of the guest vapors into the organic semiconductor.^13^

Inspired by postsynthetic modifications involving the insertion of iodine molecules into insulating MOFs to enable charge transfer along the framework, Lee et al.^12^ investigated the electrical response of I_2_-infiltrated Co-based MOF thin films for potential applications in solar harvesting devices. Thin films of the cobalt(II) 2,6-naphthalendicarboxylic acid [Co_3_(NDC)_3_DMF_4_] framework were fabricated using layer-by-layer (LBL) and doctor-blade (DB) techniques yielding thicknesses of 0.4 and 4 μm, respectively. Pore infiltration with iodine was performed by dipping the MOF thin films into an acetonitrile solution of 0.1 M I_2_ for 2 h at 50 °C. According to Hall measurements, the LBL and DB MOF thin films displayed conductivities of 10^–6^ and 10^–7^ S cm^–1^.

The same strategy was employed for 3D pillared Co_1.5_(bdc)_1.5_(H_2_bpz))·DMF·4H_2_O MOFs, which comprise tri-nuclear Co clusters of 1,4-benzenedicarboxylic acid (H_2_bdc) mixed with 3,3′,5,5′-tetramethyl- 4,4′-bipyrazole (H_2_bpz) co-ligands.^14^ Iodine loading within the MOF pores was carried out by soaking the powders in a cyclohexane solution of I_2_ for 48 h. The host–guest interaction between I_2_ molecules and the phenyl CH groups along the framework is likely to induce n → σ* charge transfer, leading to an increase in the electrical conductivity from 2.59 × 10^–9^ to 1.56 × 10^–6^ S cm^–1^.

More recently, this approach has been applied to 2D MOF structures, leading to a significant improvement in their electrical properties due to the chemical oxidation of the framework induced by the incorporation of molecular iodine. This has been observed for Cu_3_(2,3,8,9,14,15-hexahydroxytrinaphthylene)_2_ (Cu_3_(HHTN)_2_) drop-casted film FET devices^10^ and Cu_3_(TABTO)_2_ pellets (where TABTO = 1,3,5-triamino-2,4,6-benzenetriol),^15^ whose conductivities were enhanced by a factor of 10^5^ and 10^9^, respectively.

Talin et al. reported the first experimental observation of thermoelectric properties in a MOF thin film with HKUST-1 being selected as a possible candidate for TE applications.^16^ The electrical insulating nature of this MOF was overcome by introducing TCNQ (tetracyanoquinodimethane) guest molecules which served as a pathway for charge transfer by bridging neighboring metal centers. The thermoelectric properties of Ni_3_(HITP)_2_ were investigated by Dincă and co-workers.^17^ The electrical conductivity and the Seebeck coefficient of this framework at room temperature were found to be 58.8 S cm^–1^ and −11.9 μV K^–1^, respectively. The high electrical conductivity of this MOF led to a figure of merit of 1.19 × 10^–3^ at ambient conditions. This value is much higher than that reported for TCNQ@HKUST-1. Although the figure of merit is still too low for practical applications, it is the first study of pristine conducting MOFs as potential TEGs. More recently, the thermoelectric properties of Cu-benzenehexathiol (Cu[BHT]) polycrystalline thin films fabricated by liquid–liquid interfacial synthesis were reported.^18^ The best TE performance was observed for a 400 nm thick Cu(BHT) thin film, which displayed a Seebeck coefficient, as well as electrical and thermal conductivity values of −21 μV K^–1^, 2000 S cm^–1^, and 0.24 W m^–1^ K^–1^ at 300 K, respectively. The authors suggested that the figure of merit (0.013) exhibited by this n-type conductive framework can be optimized through tailoring of its charge carrier concentration via redox or chemical doping approaches.

**Figure 1:**
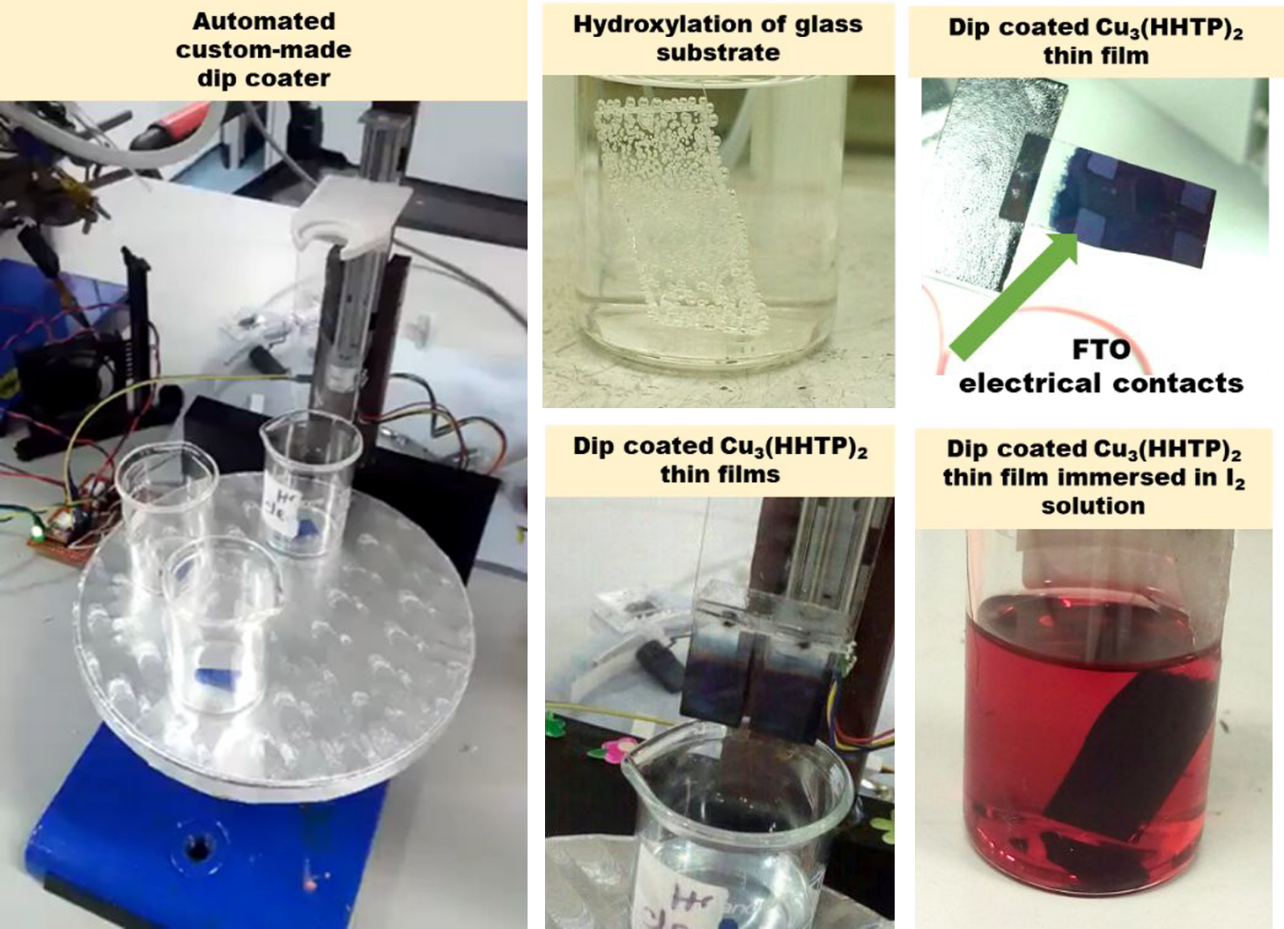
Photographs of the automated custom-made dip coater, glass substrate with etched FTO contacts immersed in piranha solution, fabricated Cu_3_(HHTP)_2_ thin films, and I_2_ loading Cu_3_(HHTP)_2_ experiment.

In this paper, we focus on the enhancement of the thermoelectric properties via iodine loading within the 2D semiconducting MOF, Cu_3_(2,3,6,7,10,11-hexahydroxytriphenylene)_2_ [Cu_3_(HHTP)_2_]. Dip coating is demonstrated as an effective technique for the preparation of semiconducting MOFs. The MOF films in this work were prepared on etched fluorine tin oxide (FTO) glass substrates by dip coating (Figure 1), and their structural integrity was investigated by grazing-incidence X-ray diffraction (GIXRD) measurements before and after guest-molecule infiltration. Electrical and Seebeck measurements were performed on the dip-coated MOF thin films after immersion in iodine solutions for different periods of time (0, 30, 60, and 90 min). Optical and chemical characterizations of the prepared iodine-loaded Cu_3_(HHTP)_2_ films were conducted via UV–vis, X-ray photoelectron spectroscopy (XPS), and Raman spectroscopy. The incorporation of iodine into Cu_3_(HHTP)_2_ dip-coated films resulted in a power factor of 0.757 μW m^–1^ K^–2^ for this framework, which is mainly attributable to the enhancement in the electrical conductivity of the material.

## 2 Results and Discussion

The growth of Cu_3_(HHTP)_2_ thin films has previously been carried out by a number of methods including the Langmuir–Blodgett technique,^19^ immersion of a graphite-patterned polymeric film into the MOF synthesis solution,^20^ and a spray-based layer-by-layer liquid-phase epitaxial method.^21^ Dip coating is considered to be a low cost technique which is used for uniform coatings over large areas regardless of the substrate geometry. The fabrication of MOF films by dip coating is considered to be a layer-by-layer (LBL) growth method.^22,23^ The substrate surface is first functionalized in order to provide anchoring groups which serve as nucleation sites by mimicking the terminal groups present in the organic linkers (e.g., −OH groups). The functionalized substrate is then immersed in a solution containing metal ions which bind to the functionalized surface, followed by a rinse step. The substrate is immersed in the ligand solution and rinsed once again. This process is repeated *n* times as desired, and the thickness of the film increases linearly as a function of the number of dipping cycles. Due to the individual dipping steps of the substrate conducted in metal salt and ligand precursor solutions with no extra additives, the resultant deposited MOF films are homogeneous and structurally well ordered. Thus, dip coating offers a more accessible synthetic route to configure MOFs as thin films, even with the possibility to obtain preferably oriented films when using the appropriate surface anchoring functional groups.^22,24^

In this work, dip-coated Cu_3_(HHTP)_2_ thin films were fabricated using an automated custom-made dip coater. The concentration of the precursor solutions comprising Cu(OAc)/EtOH and HHTP/EtOH was 0.01 mg mL^–1^. The etched FTO glass substrate was first immersed in the metal precursor solution (dwell time = 20 s), followed by a rinsing step in an ethanol solution to remove the unreacted product (dwell time = 5 s). The substrate was then immersed in the ligand precursor solution (dwell time = 40 s) followed by a rinsing step. The sequence of the dipping steps was repeated up to 50 times. The FTO glass substrates were subjected to a surface chemical treatment prior to film formation, which resulted in free −OH groups being anchored to the substrate surface that served as potential MOF nucleation centers.^25^ These functional groups mimic the hydroxyl groups present in the ligand and allow direct coordination of the metal ions to the surface and facilitate the growth of MOF crystals.

**Figure 2:**
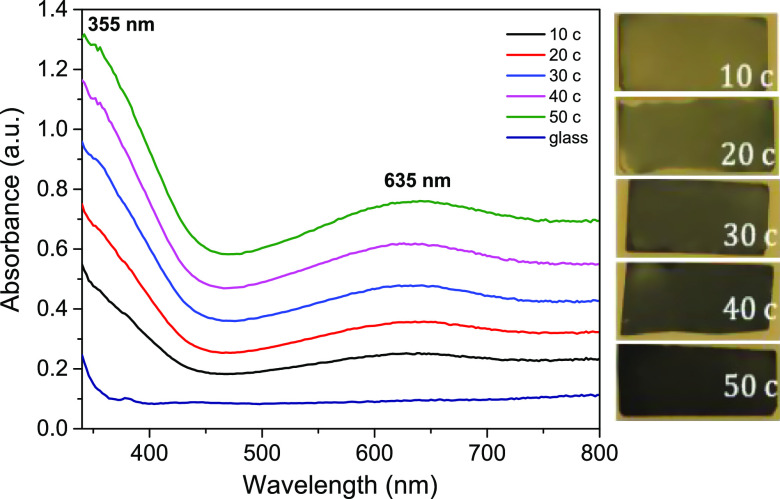
Absorbance spectra of the dip-coated Cu_3_(HHTP)_2_ MOF thin films after 10, 20, 30, 40, and 50 dipping cycles and their respective photographs. Concentration of both precursor solutions was 0.01 mg mL^–1^.

The first observation during the dip-coating process was the gradual darkening of the substrates which suggested the successful growth of the Cu_3_(HHTP)_2_ films. UV–vis absorbance spectra of the Cu_3_(HHTP)_2_ MOF thin films were carried out at room temperature (Figure 2). An increase in the absorbance values of the MOF films is observed as the number of dipping cycles increased, confirming an expected increase in the thickness of the films. Each spectrum exhibits two absorption maxima at 355 and 635 nm which may be attributed to the π → π* transition of the catecholate ligand due to the presence of its highly conjugated aromatic rings and the d–d transition of the d^9^ (Cu^2+^) cation within the framework, respectively.^26^

The optical band gap of the dip-coated MOF films after 50 dipping cycles was calculated from Tauc plots (Figure S1). The background spectrum was subtracted from the experimental data before UV–Vis band analysis. The absorption coefficient (α) is obtained from the equation  where *t* represents the thickness of the deposited film, *I*_t_, the intensity of the transmitted light, and *I*_0_ is the intensity of the incident light, respectively. The optical band gap of the material is calculated using the expression (αhγ)^1/*n*^ = *C*(*h*γ – *E*_g_), where *h* is Planck’s constant, *C* is the proportionality constant, γ is the frequency of light, *E*_g_ is the band gap energy, and *n* = 1/2 for direct allowed transitions. The band gap of the dip-coated Cu_3_(HHTP)_2_ films was obtained by extrapolating the linear portion of the curve to the photon energy intercept giving a value of 2.7 eV, confirming the semiconducting nature of the framework. This value is in agreement with the band gap of 2.68 eV for Cu_3_(HHTP)_2_ in the bulk calculated from temperature-dependent electrical data and an Arrhenius plot reported in our previous work.^27^

The as-deposited MOF films were immersed in solutions of iodine dissolved in dichloromethane (2 mg mL^–1^) for 30, 60, 90, and 120 min and subsequently characterized by GIXRD and UV–vis spectroscopy, and their thermoelectric properties are determined.

**Figure 3:**
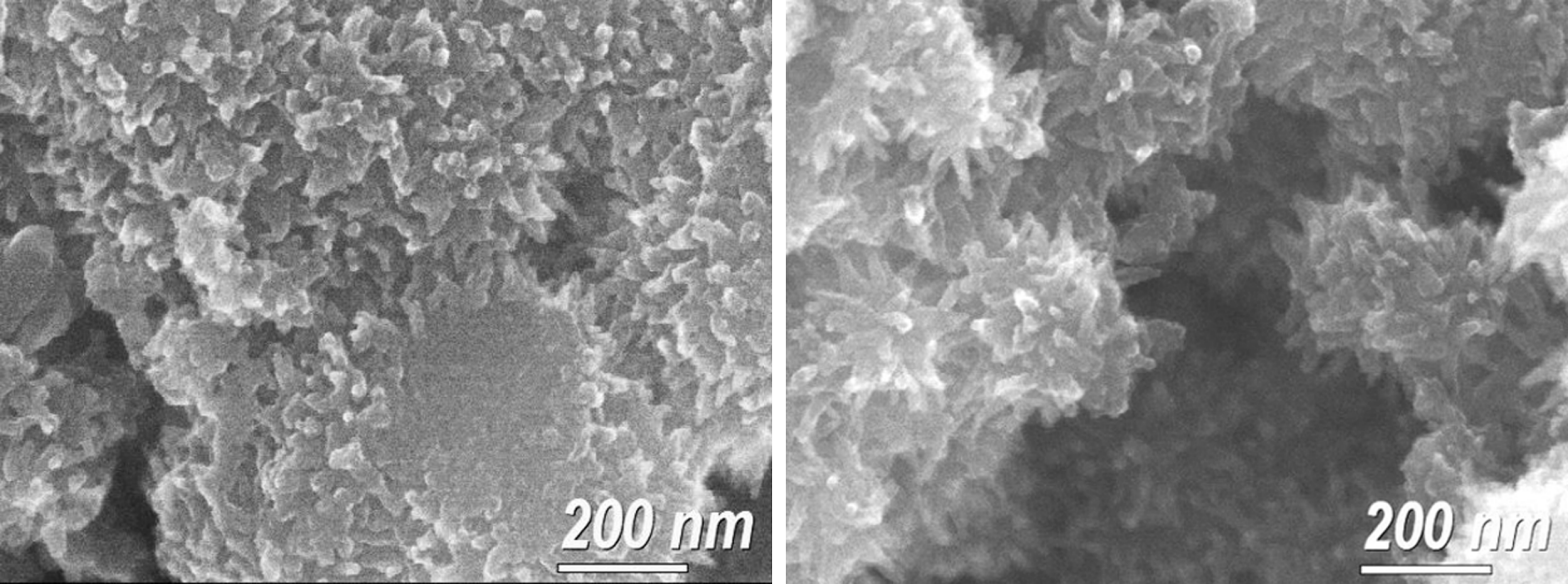
SEM micrographs of dip-coated Cu_3_(HHTP)_2_ thin films after 50 dipping cycles.

In the initial stages, the MOF films prepared by dip coating showed the formation of small clusters which were composed of individual atoms or molecules on the substrate surface.^28^ These coalesced to form a continuous film in which the thickness varies with time. Contrary to the coating method where the substrate is immersed into a solution containing the dispersed MOF powder, the layer-by-layer technique leads to a rigid anchoring of the MOF crystallites onto the surface. The grown MOF thin films prepared in this work comprised agglomerated particles with irregular shapes and an average size of ∼332 nm. The surface roughness of the dip-coated Cu_3_(HHTP)_2_ films appears to increase with the number of dipping cycles, which is attributed to an increase in the number and size of the agglomerated particles comprising the films as suggested by microstructural characterization (Figure S2). High-resolution SEM images of Cu_3_(HHTP)_2_ MOF thin films prepared by 50 dipping cycles and a concentration of 0.01 mg mL^–1^ are shown in Figure 3. The analysis revealed that the deposited MOF films exhibited nanostructured features.

A qualitative adhesive tape-peeling test was conducted on the MOF film deposited after 50 dipping cycles. It appeared that the outer layer of the MOF film was weakly bound to the substrate (Figure S3), but that a uniform film remained on the substrate, indicating that the first grown layers are rigidly attached to the substrate due to the hydroxylated glass surface.

**Figure 4:**
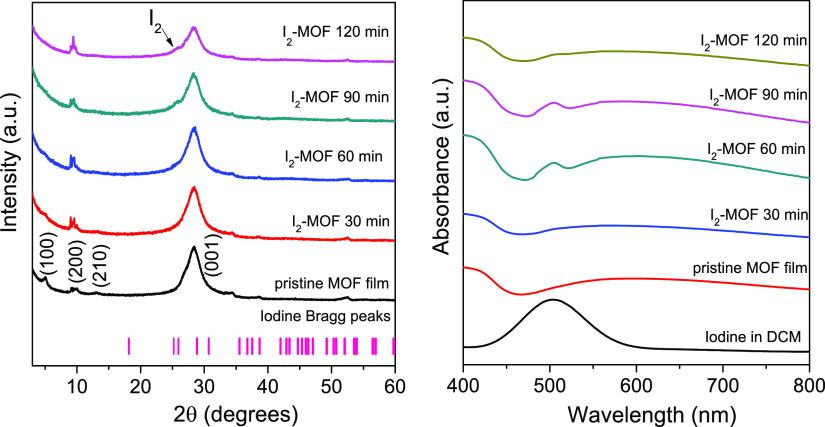
GIXRD patterns (left) and absorbance spectra of as-deposited and I_2_-loaded Cu_3_(HHTP)_2_ thin films (right).

A comparative structural characterization between pristine and iodine-loaded dip-coated Cu_3_(HHTP)_2_ films revealed broadening and slight shift to lower 2θ angles in the peak positions of the (100) and (002) crystal planes, respectively (Figure 4-left, Figure S4). The calculated XRD pattern of iodine (COD No. 1010091) is shown for clarity. The (100) peak of Cu_3_(HHTP)_2_ is ascribed to the *ab* planes being aligned with the long axis of the MOF pores; thus, the broadening of this diffraction peak indicates the distortion of the in-plane arrangement due to the incorporation of I_2_ within the MOF cavities.^10^ In addition, as suggested by the decrease in intensity of the 002 peak, a disorder in the stacking of MOF sheets along the *c* direction also occurs after I_2_ infiltration.^29^ The peak shoulder located at 25.6° for the I_2_-loaded MOF thin films is indicative of the presence of iodine, which increases in intensity with longer immersion times.^30^ In a previous report,^10^ upon the incorporation of iodine in Cu(2,3,8,9,14,15-hexahydroxytrinaphthylene) [Cu_3_(HHTN)_2_], a framework composed of a redox active skeleton with a 2.5 nm pore size, the broadening of the (100) peak was assumed to be due to the formation of I_3_^–^ chains within the pores causing reordering of the MOF layers. We speculate that a similar mechanism is operational for the Cu_3_(HHTP)_2_ system due to its similarity with the highly conjugated and redox active ligand present in the Cu_3_(HHTN)_2_ MOF. To validate the oxidation of the Cu_3_(HHTP)_2_ framework and the formation of I_3_^–^ ions, further Raman and XPS studies were conducted.

Optical characterization showed the appearance of an absorption band corresponding to molecular I_2_ with a maximum peak intensity located at 502 nm (Figure 4, right), which is first observed after an immersion time of 60 min. However, the intensity of this band decreases when the MOF film is re-immersed in the iodine solution to complete the 120 min period. Since the MOF films were not evacuated prior to I_2_ loading experiments and due to the high affinity to adsorb moisture from the environment exhibited by Cu_3_(HHTP)_2_,^31^ the absence of the I_2_ absorption band after 30 min immersion time is presumably due to the low exchange rate of water molecules within the MOF cavities replaced by molecular iodine. The less intense I_2_ absorption band after the 120 min immersion period may suggest that an equilibrium is reached, which may or may not correspond to the complete filling of the pores.

**Figure 5:**
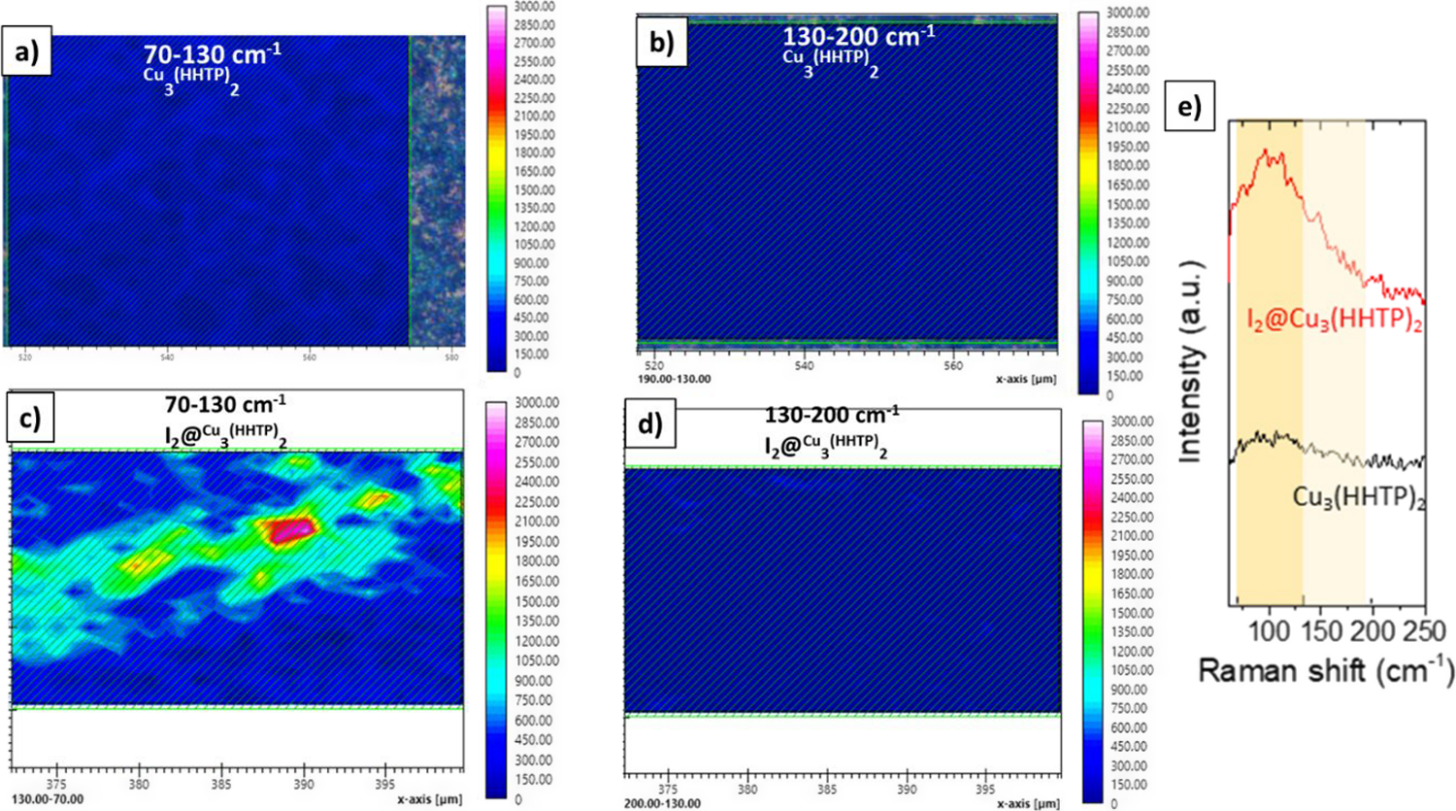
Raman mapping of pristine dip-coated Cu_3_(HHTP)_2_ film (a,b) and after iodine infiltration (c,d) with their respective Raman spectra (e).

Raman spectroscopy coupled with integral intensities mapping was conducted in order to determine the iodine species adsorbed within the Cu_3_(HHTP)_2_ cavities after 30 min of being immersed in iodine solution (Figure 5). The pristine dip-coated Cu_3_(HHTP)_2_ showed no bands in the region of interest between 70–130 and 130–200 cm^–1^ due to the absence of I_2_ (Figure 5a,b). After the MOF film was loaded with iodine, a Raman band centered at 105 cm^–1^ appears which is attributed to linear symmetric iodide anion I_3_^–^.^32^ The formation of triiodide anion species presumably occurs due to the charge transfer between iodine and the highly conjugated Cu_3_(HHTP)_2_ framework, leading to its partial oxidation.^33^ This phenomenon could explain the higher electrical conductivity of the iodine-loaded MOF film compared to the pristine film. In addition, neutral I_2_ is not found in the MOF film as suggested by the absence of a band and integral intensities in the 130–200 cm^–1^ region (Figure 5d,e). This finding is in agreement with XRD characterization data where no distinct diffraction peaks corresponding to bulk iodine were observed. The distribution of triiodide in the MOF film was elucidated by Raman mapping. The integral intensity in the range of 70–130 cm^–1^ for the MOF film immersed for 30 min in I_2_ solution showed the partial inclusion of I_3_^–^ within the MOF pores, suggesting that iodine capture occurs slowly at this stage (Figure 5c).

**Figure 6:**
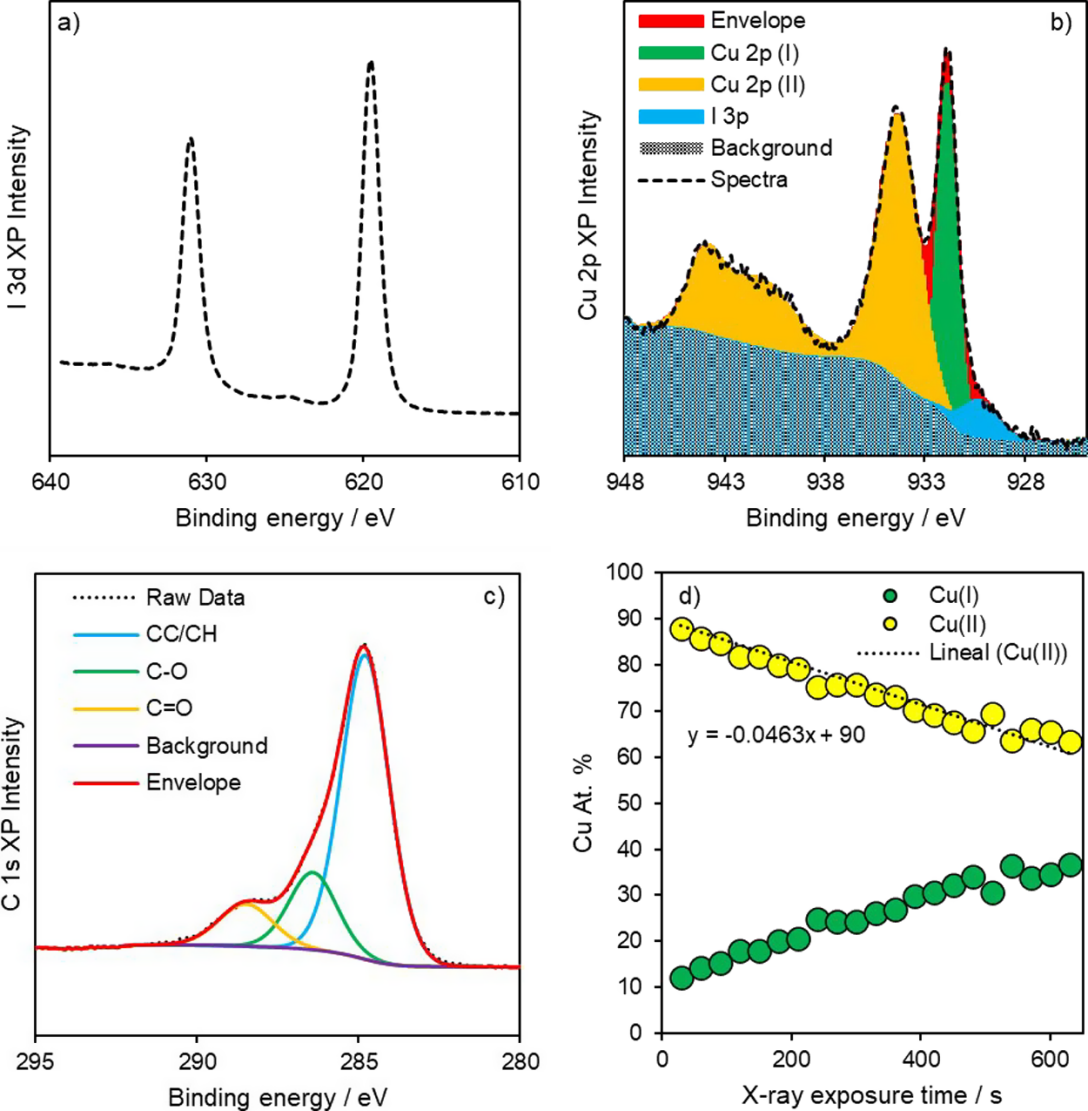
XPS spectra of I 3d (a), Cu 2p infiltration (b), and X-ray reduction profile of Cu valence states (c) as a function of exposure time (d). (c) C 1s regions for Cu_3_(HHTP)_2_ after iodine loading.

XPS analysis was performed in order to elucidate the mechanism of binding of I_2_ to Cu_3_(HHTP)_2_ and determine the valence state of the Cu metal nodes. A survey spectrum revealed the presence of the expected elements while quantification indicated an I–Cu ratio of 1:1 and a C–O ratio of approximately 3:1, in line with the ligand structure (Figure S5). High-resolution analysis was performed at a pass energy of 40 eV to enable identification of the electronic configuration of the metal and iodine (Figure 6). In order to assess the stability of the sample under the beam (and potentially under the required charge neutralization conditions,^34^ the Cu valence state as a function of X-ray exposure time was recorded (Figure S6) and spectral composition deconvoluted into a linear function (Figure 6d). In the 3d graph, the creation of a large peak at ∼931 eV associated with Cu(I) or Cu(0) arises as X-ray exposure increases, while the broader peak at higher binding energy diminishes. Analysis of the Cu LMM Auger (Figure S7) following a full irradiation period revealed a modified Auger parameter (*a*′) of 1849 eV, which is consistent with the presence of Cu(I) species.^35^ In order to attempt to circumvent this rapid reduction under even soft X-ray conditions, 20 single spot spectra were obtained on a short dwell time and averaged to reduce spectral noise. The deconvolution of this (Figure 6b) determined that the majority of the Cu is in the form of Cu(II), as evidenced by the characteristic satellite features at ∼940 eV.^35^ The determined ratio of Cu(II) to Cu(I) was found to equate to ∼85% Cu(II), which indicates that the system potentially exists at ∼90% Cu(II) minimum according to our X-ray reduction profiles (Figure 6d). Iodine speciation was determined through analysis of the I 3d region only (Figure 6a) since there exists a significant overlap between O KLL and I LMM Auger peaks which render accurate peak positioning troublesome. the peak maxima of the I 3d_5/2_ emission was found to be 619.5 eV, which is consistent with that of I_3_^–^.^36^ The presence of I_3_^–^ indicates the presence of triiodide anions and suggests the formation of a charge transfer complex between the aromatic π-electrons from the MOF framework and iodide chains.^10,37^

**Figure 7:**
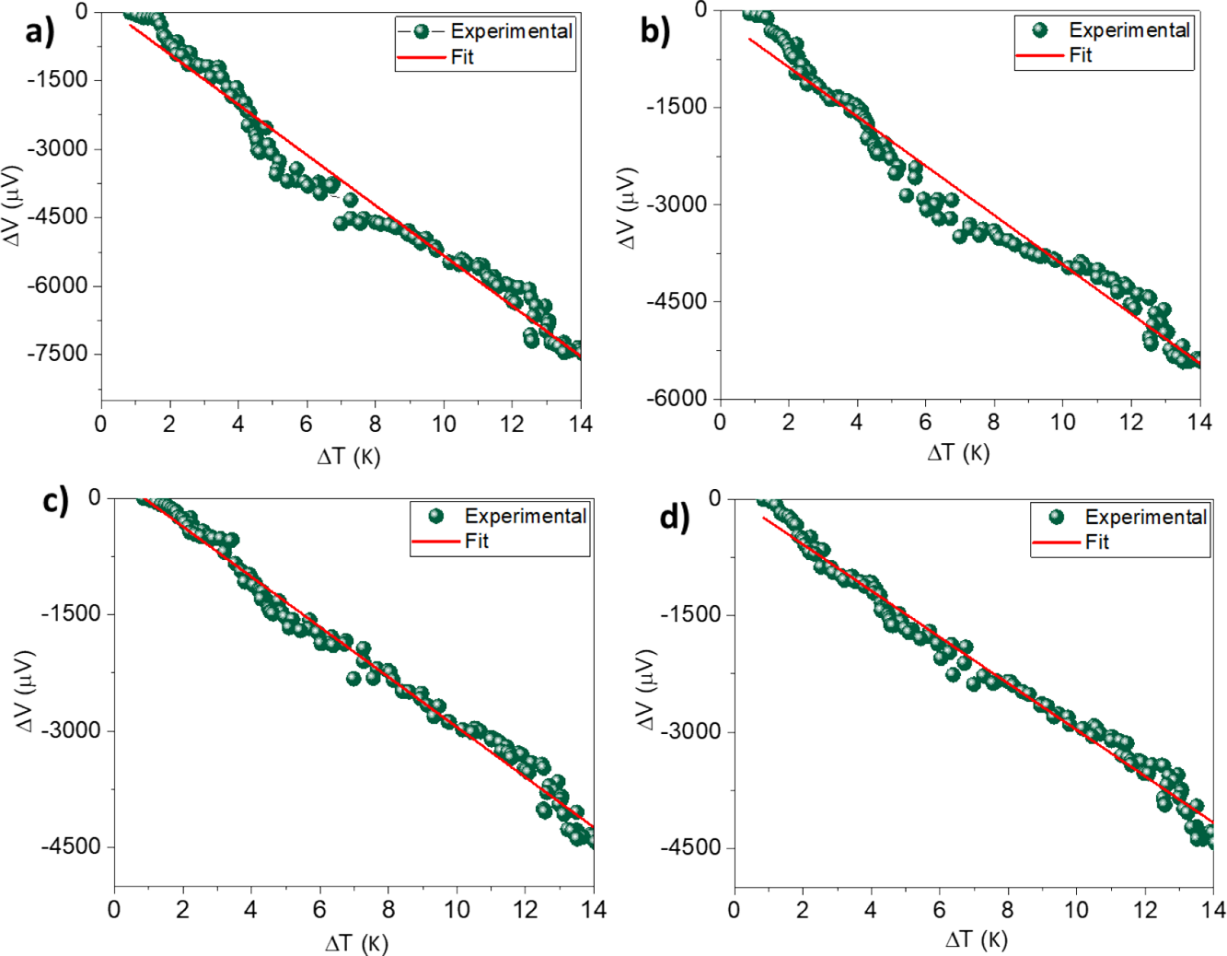
Seebeck voltage measured as a function of temperature difference for (a) pristine dip-coated Cu_3_(HHTP)_2_ film and after being immersed in iodine solution for (b) 30 min, (c) 60 min, and (d) 90 min.

**Figure 8:**
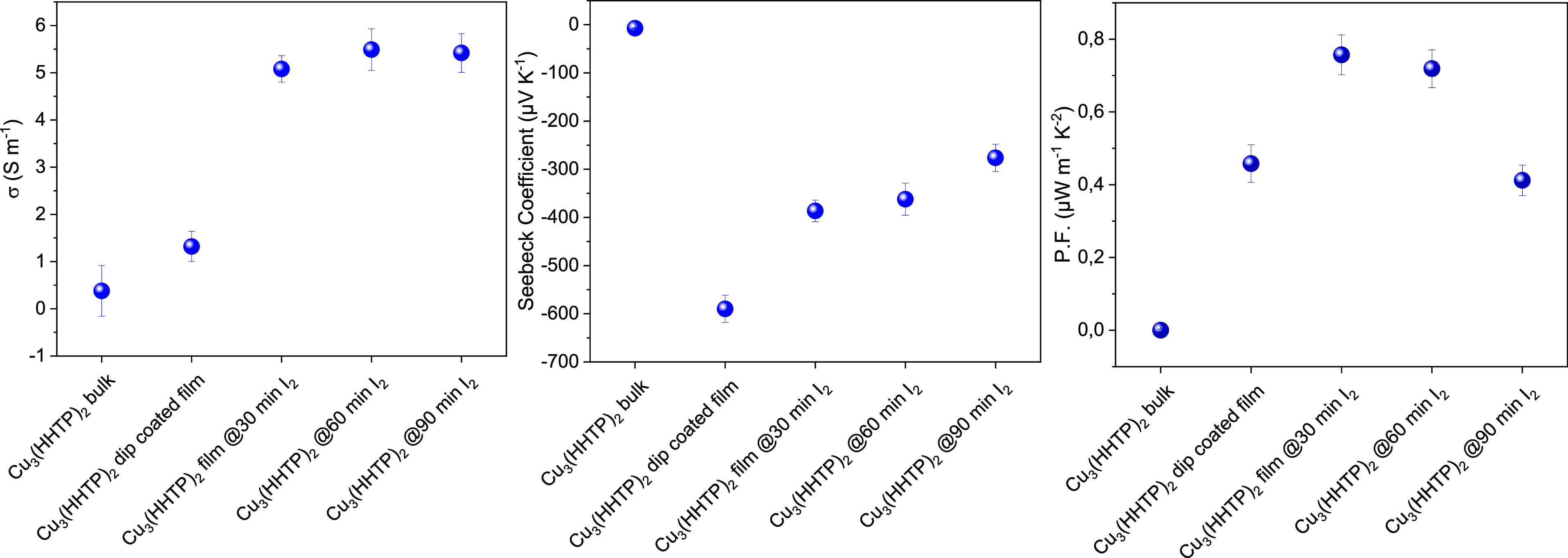
Thermoelectric performance for bulk and dip-coated Cu_3_(HHTP)_2_ thin films (before and after iodine infiltration).

The Seebeck coefficient was determined by measuring the voltage drop across the samples with two probes whilst applying a temperature gradient, as shown in Figure 7. Good Ohmic contact was confirmed by obtaining linear current versus voltage response (cf. Figure S8). The Seebeck coefficient was calculated from the slope of the linear fit (red curve) to the Seebeck voltage versus temperature difference curves. A maximum Seebeck coefficient of −589 μV/K was obtained for the pristine dip-coated films of Cu_3_(HHTP)_2_. These results were found to be reproducible and have been verified for three other samples that were prepared under identical conditions (cf. Table S2 in the ESI). The negative sign of the Seebeck coefficient indicates that the majority of carriers within this MOF is n-type. This is in agreement with what we have measured for the bulk-pressed pellets and electrodeposited thin films of Cu_3_(HHTP)_2_ which were also found to exhibit n-type semiconducting behavior.^27^ The main difference, however, is the magnitude of the Seebeck coefficient measured for the dip-coated MOF films and the pressed bulk pellet, even though we previously observed an increase in the Seebeck coefficient for electrodeposited thin films to −121.4 μV/K.^27^ To investigate the higher value of the Seebeck coefficient, we performed Hall effect measurements to determine the electrical conductivity and the charge carrier density and included SEM micrographs (cf. Figure S9, ESI) that reveal the surface morphology of the pressed bulk MOF pellet and the dip-coated films. By closely inspecting the SEM micrographs of the surface of the pressed bulk pellet and comparing this to the as-prepared dip-coated films, we can observe noticeable cracks in the bulk pellet which can explain the much lower electrical conductivity as opposed to the much higher electrical conductivity observed for the dip-coated films, as can be seen in Figure 8 and Table S1. The presence of cracks in the pellet also did not allow us to accurately measure the charge carrier density in the bulk pellet, and we attribute the much lower Seebeck coefficient to the possibility that the bulk pellet is in fact highly doped compared to the as-prepared dip-coated films which can explain the much lower Seebeck coefficient. The other noticeable feature from the SEM micrographs is the presence of nanostructured features within the dip-coated films which may also be a contributing factor to explain the much higher Seebeck coefficient. In other studies on similar MOFs,^17^ the processing conditions were found to play a major role in enhancing the value of the Seebeck coefficient between bulk-pressed pellets and thin films. After iodine doping, the Seebeck coefficient was found to decrease to −386.4 μV/K whilst the electrical conductivity increased to 5.08 S m^–1^. Molecular doping with iodine has been reported as enhancing the electrical conductivity in other MOF systems.^17^ The carrier density after doping changed from −2.66 × 10^18^ to −8.69 × 10^17^ cm^–3^ which can be explained in terms of oxidative molecular doping by iodine that decreased the number of n-type carriers. XPS confirmed the formation of an I_3_^–^ species and the presence of Cu^2+^ which provides further evidence for oxidative doping via iodine which is in agreement with other studies as well.^10^

As can be seen from Figure 8 (Table S1), the Cu_3_(HHTP)_2_ MOF film immersed for 30 min in iodine led to a record power factor of 0.757 μW m^–1^ K^–2^ for this framework. Although this value is much higher than those of other thermoelectric MOF systems with infiltrated guest molecules such as TCNQ@HKUST-1^16^ with an experimental power factor (PF) of 0.057 μW m^–1^ K^–2^, pristine Ni_3_(hexaaminotriphenylene)_2_ frameworks possess the highest thermoelectric performance reported so far with a PF of 8.31 μW m^–1^ K^–2^.^17^ Our PFs are lower due to a much lower electrical conductivity which may be explained in terms of the metallic band structure for the Ni_3_(hexaaminotriphenylene)_2_ framework.

Lee et al. investigated the charge transport properties of HKUST-1 thin films fabricated on glass substrates by doctor-blading (DB) and layer-by-layer (LBL) techniques with thicknesses of ∼4 and ∼0.5 μm, respectively.^38^ Iodine loading was conducted by dipping the MOF thin films in an acetonitrile solution of 0.1 M I_2_ for 2 h at room temperature. Electrical measurements showed a 100-fold increase in the conductivity of HKUST-1 thin films compared to the pristine framework. The charge transport mechanism is attributed to the confinement of I_2_ molecules within the pores, leading to intermolecular interactions between these guest molecules and the aromatic rings resulting in a cooperative electrical conductivity increase of this material. Various examples on the enhancement of the electrical properties of different MOF systems before and after iodine infiltration have been reported in the literature and are summarized in Table S3.

## 3 Conclusions

The main advantage of the synthesis of MOFs by the dip coating method is its simplicity as neither robust equipment nor critical synthesis conditions are needed. Thermoelectric properties of dip-coated Cu_3_(HHTP)_2_ films with and without iodine loading are reported for the first time. The incorporation of I_2_ within the MOF film resulted in a noticeable increase in the electrical conductivity, leading to a decrease in the Seebeck coefficient (as expected due to the interdependency of these two physical properties). We report record PFs for pristine and I_2_-loaded Cu_3_(HHTP)_2_ thin films with values of 0.458 and 0.757 μW m^–1^ K^–2^, respectively. These PFs are much higher than the bulk equivalent, and the only example of a thermoelectric MOF loaded with guest molecules reported to date is TCNQ@HKUST-1. These findings demonstrate that tuning of the thermoelectric performance of Cu_3_(HHTP)_2_ can be achieved through material processing and guest-molecule infiltration. Overall, these findings suggest that MOFs could be considered as the new generation of thermoelectric materials. The electronic behavior of MOFs is influenced by the transition metal d-electrons and their interaction with the molecular orbitals of the organic linkers, and electrical conductivity can be enhanced by the introduction of guest molecules into the pores to increase the charge mobility along the framework or by the selection of redox active organic molecules as building blocks.

## 4 Experimental Methods

### 4.1 Materials

**Figure 9:**
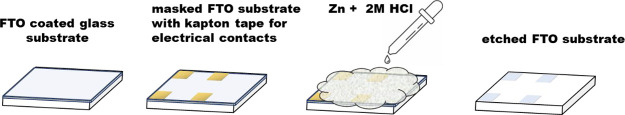
Preparation of FTO electrical contacts on glass substrates.

Copper(II) acetate monohydrate and the ligand 2,3,6,7,10,11-hexahydroxytriphenylene hydrate were used as chemical precursors. In order to monitor variations in electrical conductivity and the Seebeck coefficient, dip-coated Cu_3_(HHTP)_2_ thin films (1 cm × 1.5 cm) were prepared on glass substrates with fluorine-tin oxide (FTO) contacts (Figure 9). The preparation of the FTO glass substrates (FTO layer 1 μm thick, 15 Ω/cm^2^) before dip coating was conducted by masking the four corners of the substrate with Kapton tape so these would serve as electrical contacts. The remaining unmasked FTO areas of the substrate were etched by rubbing zinc powder onto the glass surface using a spatula for 5 min followed by the dropwise addition of 2 M HCl. The Zn/HCl mixture was in contact with the substrate surface for 10 min. Finally, the glass substrates were rinsed with copious DI water and the Kapton tape was removed from the substrate corners. Alternatively, sonication can be conducted to remove any potential Zn residues on the substrate surface. A zero electrical response from the etching area was verified by using a multimeter, which indicates the successful removal of the unmasked FTO layer. Hydroxylation of the prepared glass substrates with FTO contacts was conducted by immersing them in a piranha solution (H_2_SO_4_/H_2_O_2_ ratio 6:4) for 1 h at 50 °C. After this, the substrates were rinsed with water and ethanol and left to dry at room conditions.

**Figure 10:**
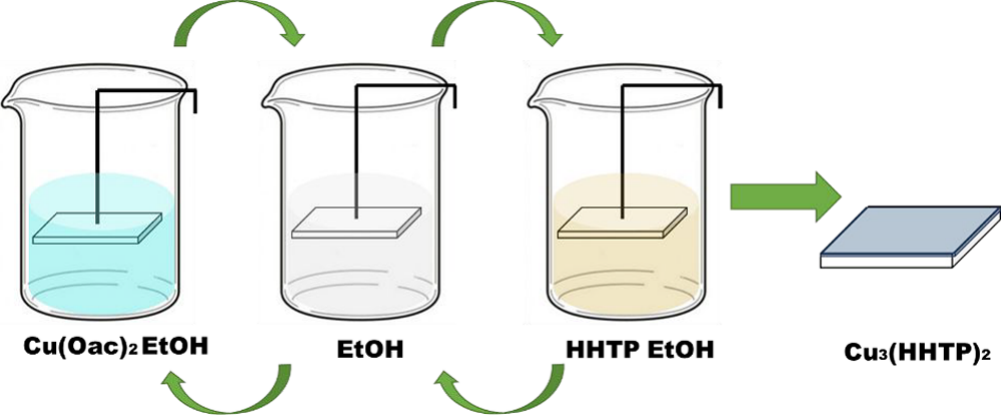
Immersion step sequence to fabricate Cu_3_(HHTP)_2_ thin films by dip coating.

Glass substrates were mounted on the dip coater holder and vertically immersed in the metal precursor solution (dwelling time = 20 s), followed by a washing step in ethanol solution (dwelling time = 5 s) to remove the unreacted product and ensure the uniform growth of the film; then, the substrates were immersed in the ligand precursor solution (dwelling time = 40 s) followed by the last washing step (dwelling time = 5 s), as shown in Figure 10. This process was repeated 50 times.

Iodine loading experiments were carried out by dissolving 20 mg of iodine in 10 mL of dichloromethane (DCM). This solvent was chosen due to the high solubility of iodine and its high volatility, so the treated MOF thin film samples can be characterized almost immediately after removing them from the I_2_ solution.

## Supporting Information Available

Characterization; energy band gap of dip-coated Cu_3_(HHTP)_2_ film; SEM micrographs of Cu_3_(HHTP)_2_ thin film; Cu_3_(HHTP)_2_ film after the adhesion test; PXRD patterns of pristine Cu_3_(HHTP)_2_ film and after I_2_ loading; survey spectrum of dip-coated Cu_3_HHTP)_2_ thin film; XPS analysis of the Cu valence state; Cu LMM Auger spectrum for dip-coated Cu_3_(HHTP)_2_; *I*–*V* curves of pristine and iodine-doped Cu_3_( HHTP)_2_ films; thermoelectric performance for bulk and Cu_3_(HHTP)_2_ thin film (Table S1); SEM micrographs of Cu_3_(HHTP) pressed pellet and dip-coated film; thermoelectric data for four dip-coated Cu_3_(HHTP)_2_ films (Table S2); and comparison of electrical properties of MOFs before and after I_2_ loading reported in the literature (Table S3) (PDF)
