# Supplementary material for: Enhanced Thermoelectric Properties of a Semiconducting Two-Dimensional Metal–Organic Framework via Iodine Loading
Source: ACS Appl Mater Interfaces. 2023 Jan 23;15(4):5478–86. doi: 10.1021/acsami.2c20770 (PMC9906625; doi:10.1021/acsami.2c20770)
Supplement: Supplementary file 1 — am2c20770_si_001.pdf [file am2c20770_si_001.pdf]

Supporting information

Enhanced thermoelectric properties of a semiconducting 2D Metal-Organic Framework via iodine loading

Maria Gonzalez-Juarez[a], Mark A. Isaacs[b-c], Darren Bradshaw[a] and Iris Nandhakumar*[a]*

[a]School of Chemistry, University of Southampton, Southampton SO17 1BJ, UK

[b]Department of Chemistry, University College London, London WC1H 0AJ, UK
[c]HarwellXPS, Research Complex at Harwell, RAL, Harwell Campus, Didcot, OX11 0FA, UK

Email: I.Nandhakumar@soton.ac.uk and D.Bradshaw@soton.ac.uk

Characterization

GIXRD, UV-Vis, SEM, and thermoelectric performance characterisations of dip coated $Cu_3(HHTP)_2$ thin films were conducted prior and after iodine loading as a function of immersion time (e.g., 0.5, 1, 1.5, and 2 hr). Absorbance measurements of dip coated $Cu_3(HHTP)_2$ thin films were carried out in a Perkin Elmer instrument UV-Vis-NIR S750. Grazing incidence X-ray diffraction (GIXRD) was conducted using a Rigaku Smartlab, CuKα radiation, λ=1.5406 Å. Electrical measurements were conducted using a commercial Nanometrics HL5500 Hall measuring system based on the van der Pauw technique. The electrical conductivity was measured by applying a constant current through two equidistant probes placed onto the FTO contacts on one side of the specimen and the voltage drop is measured with two other probes placed in parallel to the probes supplying the current. The distance between electrical contacts in the x (length) and y (width) direction, was 10 mm and 6 mm, respectively.

Thermoelectric measurements were carried out at ambient conditions using a Seebeck Coefficient Tester (PTM)-Wuhan Joule Yacht Technology Co. The Seebeck coefficient was determined by applying a temperature differential across the sample while measuring the voltage drop with two probes, each one located at the hot and cold side of the sample, respectively, with a separation distance of 10 mm. Morphological and thickness characterisations were carried out using a scanning electron microscope JEOL JSM-6500F.

XPS Analysis was performed using a Thermo NEXSA XPS fitted with a monochromated Al kα X-ray source (1486.7 eV), a spherical sector analyser and 3 multichannel resistive plate, 128 channel delay line detectors. All data was recorded at 19.2W and an X-ray beam size of 400 x 200 µm. Survey scans were recorded at a pass energy of 200 eV, and high-resolution scans recorded at a pass energy of 40 eV.

Electronic charge neutralization was achieved using a Dual-beam low-energy electron/ion source (Thermo Scientific FG-03). Ion gun current = 150 µA. Ion gun voltage = 45 V. All sample data was recorded at a pressure below 10^{-8} Torr and a room temperature of 294 K. Data was analysed using CasaXPS v2.3.19PR1.0. Peaks were fit with a Shirley background prior to component analysis. Lineshapes of LA (1.53,243) were used to fit components.

Figure S1. Energy band gap of dip coated $Cu_3(HHTP)_2$ film estimated from Tauc plot.

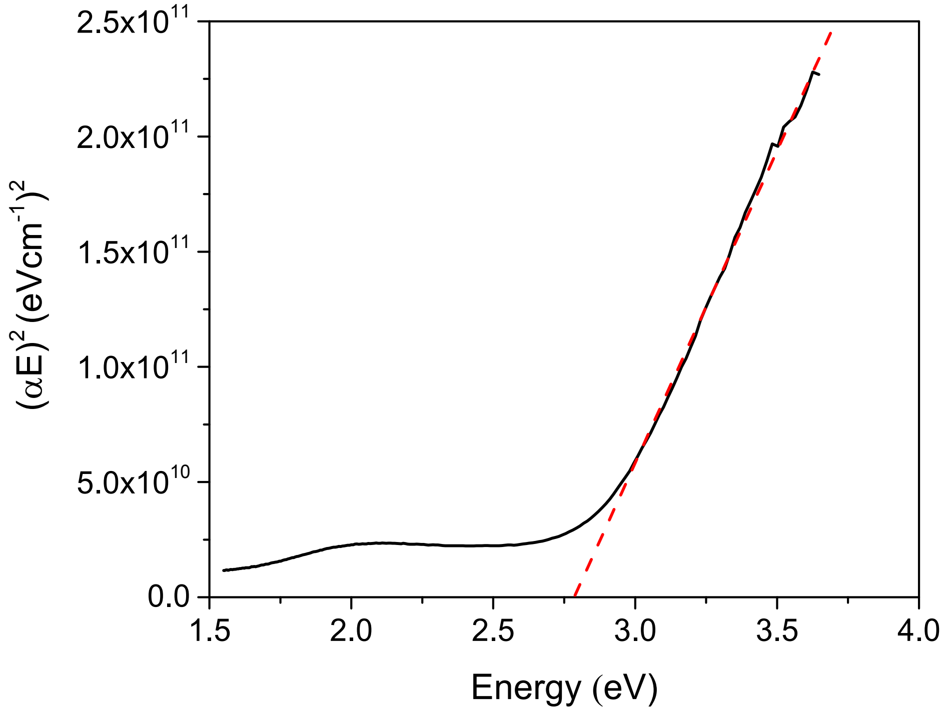

Figure S2. SEM micrographs of $Cu_3(HHTP)_2$ thin film at 10 a), 20 b), 30 c), 40 d, and 50 e-f) dipping cycles. Concentration of all precursor solutions = 0.01 mg ml^{-1}.

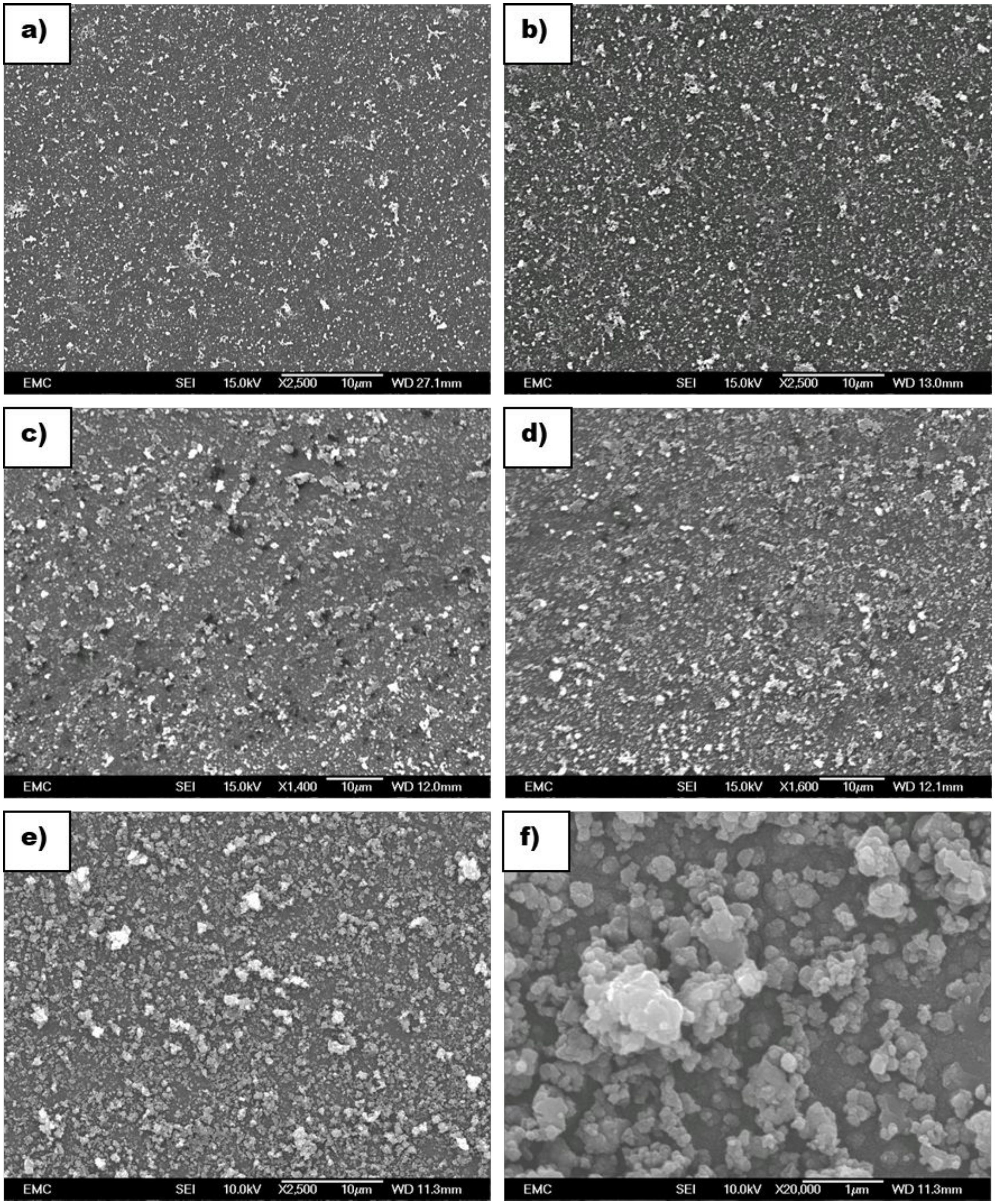

Figure S3. $Cu_3(HHTP)_2$-50c film sample after film adhesion test.

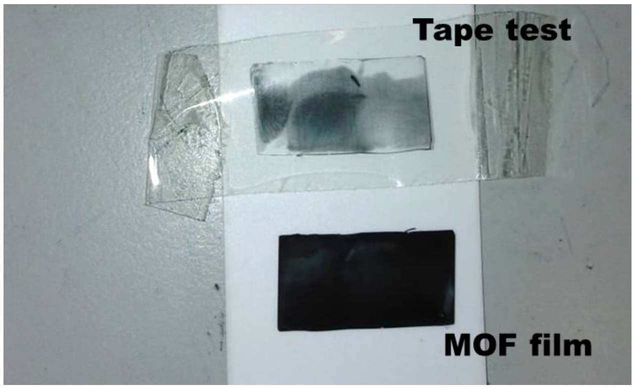

Figure S4. PXRD patterns of pristine $Cu_3(HHTP)_2$ film and after I_2 loading at different immersion time. Considerable broadening of the (100) and (002) planes after I_2 infiltration.

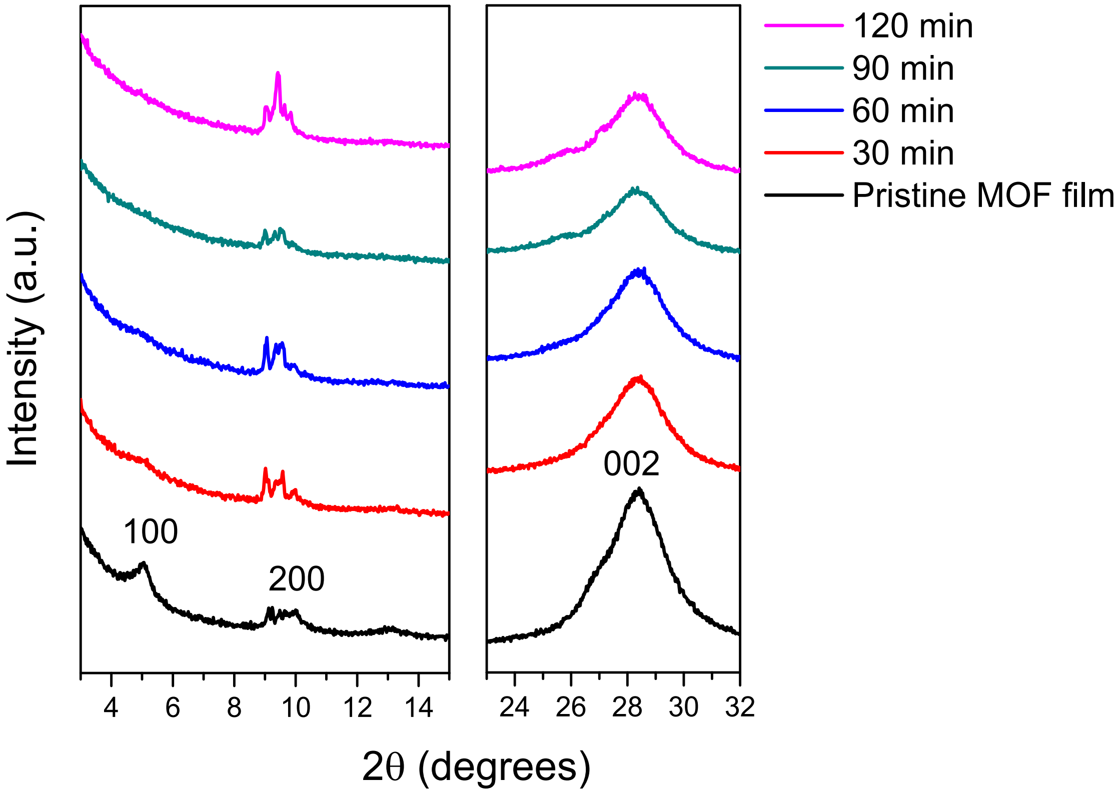

Figure S5. Survey spectrum of dip coated $Cu_3HHTP)_2$ thin film after iodine infiltration with its calculated elemental composition**.**

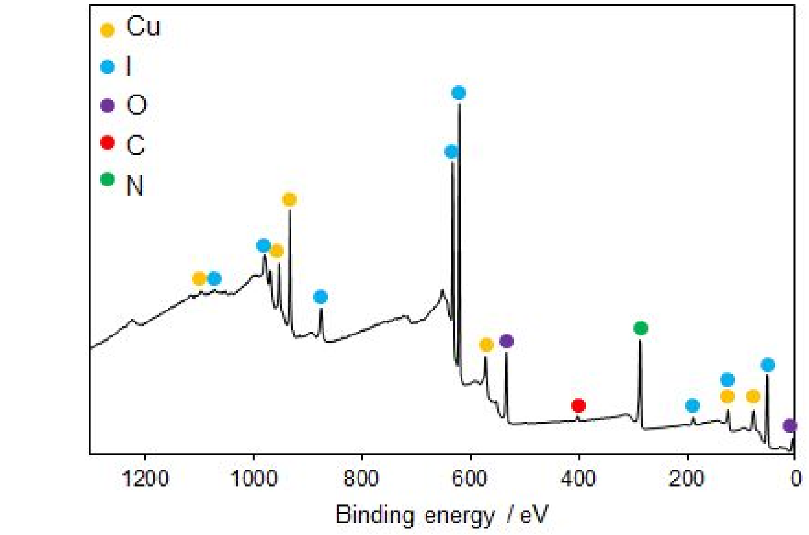

	Cu	I	N	O	C
At%	10	10.77	2.02	17.92	59.29
+/-	0.048982	0.012146	0.599892	0.229223	0.690205

Figure S6. XPS analysis of Cu valence state as a function of X-ray exposure time.

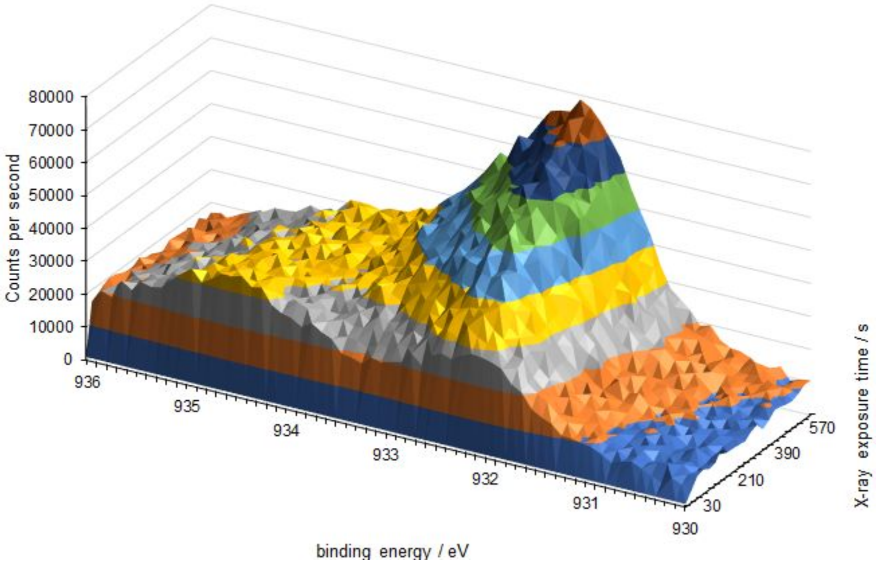

Figure S7. Cu LMM Auger spectrum for dip coated $Cu_3(HHTP)_2$ after iodine infiltration.

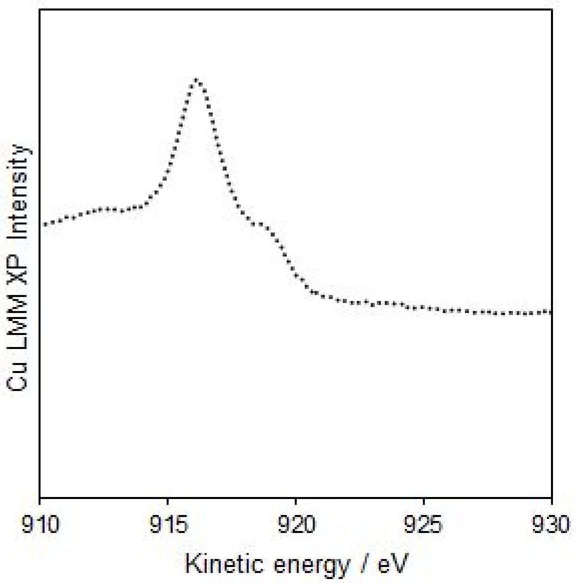

Figure S8. I-V curves of pristine $Cu_3(HHTP)_2$ dip coated (a) and after iodine infiltration for 30 min (b), 60 min (c) and 90 min (d). The electrical conductivity is obtained using the Van der Pauw method. In practice the specimen should have a high degree of symmetry and the contacts should lie on symmetry axes. Each of the four contact permutations (1-2, 2-3, 3-4, 4-1, 1-3, 2-4) is recorded by the instrument, with current reversal, and the results are tabulated as shown below. The thickness of the film sample is a parameter

a)

CONTACT CHECK

Pair	M-ohms
12	1.1e+01
23	1.3e+01
34	1.3e+01
41	1.0e+01
13	1.4e+01
24	1.2e+01

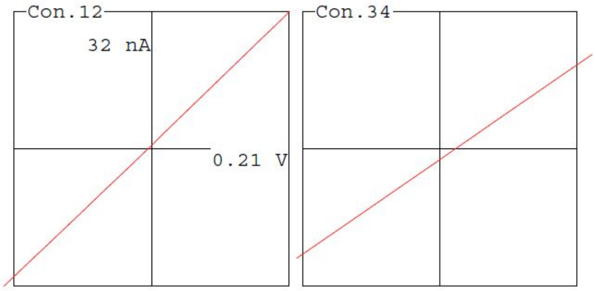

b)

CONTACT CHECK

Pair	M-ohms
12	7.6e+00
23	8.3e+00
34	7.1e+00
41	5.9e+00
13	8.9e+00
24	6.6e+00

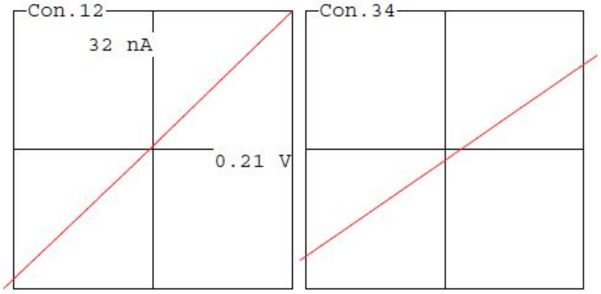

c)

CONTACT CHECK

Pair	M-ohms
12	7.6e+00
23	8.3e+00
34	7.1e+00
41	5.9e+00
13	8.9e+00
24	6.6e+00

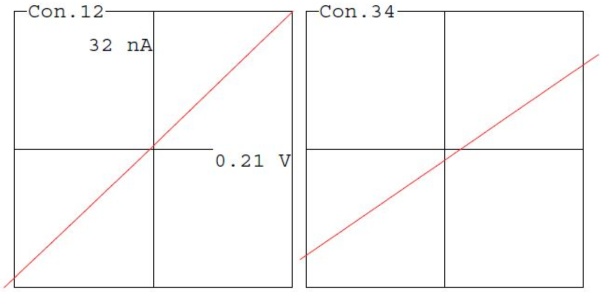

d)

CONTACT CHECK

Pair	M-ohms
12	7.6e+00
23	8.3e+00
34	7.1e+00
41	5.9e+00
13	8.9e+00
24	6.6e+00

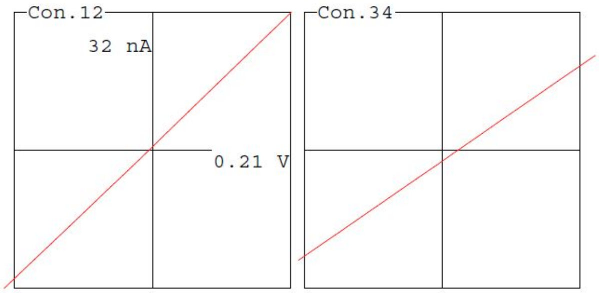

required for bulk conductivity measurements, which was found to be ~100 nm for the dip Equation 1.1 shows the calculated resistance (R) obtained for every set comprising four electrical contacts. The sheet resistance (R_s) is calculated using equations 1.2 and 1.3 with R_A and R_B being the component resistances

associated with the direction of the applied current (either along the X or Y axis, respectively). In Equation 1.4, *d* is the thickness of the sample, ρ is the bulk resistivity and σ is the bulk electrical conductivity.

$$R_{21,34} = \frac{V_{34}}{I_{21}} \quad , \quad R_{12,43} = \frac{V_{43}}{I_{12}} \qquad (1.1)$$

$$R_{32,41} = \frac{V_{41}}{I_{32}} \quad , \quad R_{23,14} = \frac{V_{14}}{I_{23}}$$

$$R_{43,12} = \frac{V_{12}}{I_{43}} \quad , \quad R_{34,21} = \frac{V_{21}}{I_{34}}$$

$$R_{14,23} = \frac{V_{23}}{I_{14}} \quad , \quad R_{41,32} = \frac{V_{32}}{I_{41}}$$

$$R_A = \frac{(R_{21,34} + R_{12,43} + R_{43,12} + R_{34,21})}{4} \; , R_B = \frac{(R_{32,41} + R_{23,14} + R_{14,23} + R_{41,32})}{4} \qquad (1.2)$$

$$\exp\left(-\pi\frac{R_A}{R_S}\right) + \exp\left(-\pi\frac{R_B}{R_S}\right) = 1 \qquad (1.3)$$

$$\rho = R_s d \quad , \quad \sigma = \frac{1}{\rho} \qquad (1.4)$$

Charge carrier densities of the samples were investigated by Hall measurements. The Hall set up consists of a magnetic field induced in the normal direction of the specimen whilst a current is supplied through the probes. Contrary to the conventional electrical measurements described earlier, here, the voltage drop is recorded by probes placed perpendicular to the current probes. The sample is tested in the positive and negative directions of the magnetic field. This configuration is done by inverting the position of the magnet, which causes the charge carriers to separate at both ends of the sample into holes and electrons generating the known Hall voltage. The dominant carrier type in the sample is deduced by the sign of the resultant Hall voltage, being negative and positive for n-type and p-type conductions, respectively.

The charge carrier density n_s (cm^{-3}) is calculated according to equation 1.5:

$$n_s = \frac{8 \times 10^{-8} IB}{q|\Sigma Vi|} \qquad 1.5$$

Where I is the supplied current (A), B is the magnetic field to which the sample is exposed (Gauss), q is the elementary charge (1.60×10^{-19} C) and ΣVi is the sum of the measured voltages at positive and negative magnetic fields.

Table S1. Summary of thermoelectric performance for bulk and $Cu_3(HHTP)_2$ thin film (pristine films with and without iodine loading).

Sample	Electrical conductivity ($S\ m^{-1}$)	Seebeck coefficient ($\mu V\ K^{-1}$)	P.F. ($\mu W\ m^{-1}\ K^{-2}$)
$Cu_3(HHTP)_2$ bulk	0.38±0.54	-7.24±0.73	2×10^{-5}±0.0032
$Cu_3(HHTP)_2$ film	1.32±0.32	-589.73±32.59	0.458±0.052
30 min I_2	5.08±0.28	-386.37±22.30	0.757±0.049
60 min I_2	5.49±0.44	-362.2±31.28	0.719±0.067
90 min I_2	5.42±0.41	-276.33±28.33	0.412±0.033

Figure S9. SEM micrographs of $Cu_3(HHTP)_2$ pressed pellet (top) and dip coated film (bottom) surfaces.

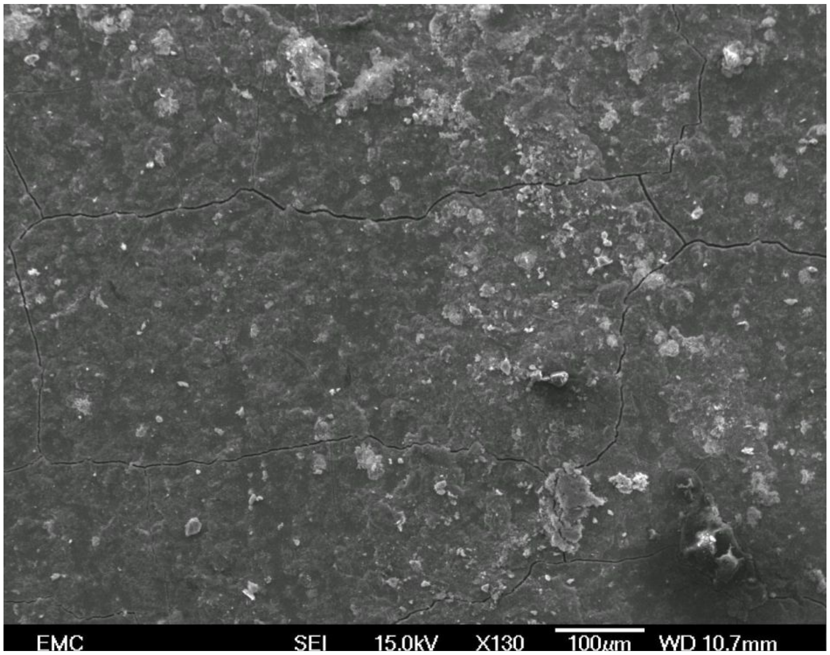

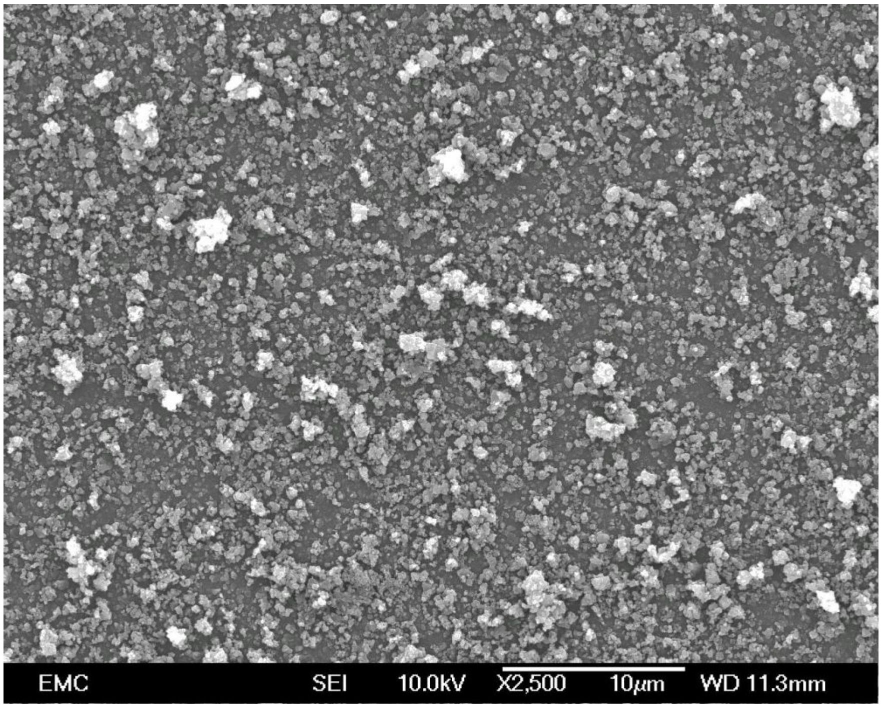

Table S2. Thermoelectric data measured from four pristine dip coated MOF films prepared under the same conditions.

	Electrical conductivity (S m^{-1})	Seebeck coefficient (μV K^{-1})	Power Factor (μW m^{-1} K^{-2})
Sample 1	1.18	-537.86	0.340
Sample 2	1.22	-565.66	0.388
Sample 3	1.32	-589.73	0.458
Sample 4	1.50	-502.2	0.385

Table S3. Comparison of electrical properties of MOFs before and after I_2 loading reported in the literature.

Framework	BET ($m^2\ g^{-1}$)	pore size (Å)	Pristine σ ($S\ cm^{-1}$)	I_2 loaded MOF σ ($S\ cm^{-1}$)	Ref.
$Cu(Ni(pdt)_2)$	385	-	1.00×10^{-8}	1.00×10^{-4}	1
$\{(Zn_3(DLlac)_2(pybz)_2)$	762.5	10. 5 A	1.65×10^{-4}	3.42×10^{-3}	2
HKUST-1	-	14 A	1.00×10^{-7}	1.00×10^{-5}	3
$Eu_4(BPT)_4(DMF)_2(H_2O)_8$	69.6	9.5 x 8.4 (pore A) 7.8 x 9.2 (pore B)	8.27×10^{-7}	2.71×10^{-5}	4
$Co_3(NDC)_3DMF_4$	-	-	-	1.88×10^{-6}	5
$(Co_{1.5}(bdc)(H_2bpz))$	148.8	3.4-6.6 A	2.59×10^{-9}	1.56×10^{-6}	6
$Ca_2(TBAPy)$	174	-	-	5.3×10^{-6}	7
MFM-300(V^{III})	-	6.7	1.70×10^{-10}	1.20×10^{-4}	8
$Cu_3(HHTN)_2$	486	25	9.55×10^{-10}	1×10^{-5}	9
$Cu_3(TABTO)_2$	33	11	1.00×10^{-9}	0.78	10

References

1. Y. Kobayashi, B. Jacobs, M. D. Allendorf and J. R. Long, Conductivity, Doping, and Redox Chemistry of a Microporous Dithiolene-Based Metal-Organic Framework, *Chem. Mater.*, **2010**, *22*, 4120–4122, DOI: 10.1021/cm101238m
2. M. H. Zeng, Q. X. Wang, Y. X. Tan, S. Hu, H. X. Zhao, L. S. Long and M. Kurmoo, Rigid Pillars and Double Walls in a Porous Metal-Organic Framework: Single-Crystal to Single-Crystal, Controlled Uptake and Release of Iodine and Electrical Conductivity, *J. Am. Chem. Soc.*, **2010**, *132*, 2561–2563. DOI: 10.1021/ja908293n.
3. D. Y. Lee, D. V. Shinde, S. J. Yoon, K. N. Cho, W. Lee, N. K. Shrestha and S. H. Han, Charge Transfer-Induced Molecular Hole Doping into Thin Film of Metal−Organic Frameworks, *J. Phys. Chem. C*, **2014**, *118*, 16328–16334, DOI 10.1021/acsami.5b04771.
4. Z. Hao, G. Yang, X. Song, M. Zhu, X. Meng, S. Zhao, S. Song and H. Zhang, A europium(III) based metal–organic framework: bifunctional properties related to sensing and electronic conductivity *J. Mater. Chem. A*, **2014**, *2*, 237–244, DOI: 10.1039/c3ta13179c.
5. D. Y. Lee, E. K. Kim, N. K. Shrestha, D. W. Boukhvalov, J. K. Lee and S. H. Han, Charge Transfer-Induced Molecular Hole Doping into Thin Film of Metal−Organic Frameworks, *ACS Appl. Mater. Interfaces*, **2015**, *7*, 18501–18507, DOI: 10.1021/acsami.5b04771.
6. G. P. Li, K. Zhang, H. Y. Zhao, et al., Increased Electric Conductivity upon I2 Uptake and Gas
7. Sorption in aPillar-Layered Metal–Organic Framework *ChemPlusChem* **2017**, *82*, 716 –720, DOI: 10.1002/cplu.201700063.
8. A. Gładysiak, T. N. Nguyen, M. Spodaryk, J. H. Lee, J. B. Neaton, A. Züttel and K. C. Stylianou, Incarceration of Iodine in a Pyrene-Based Metal–Organic Framework, *Chem. Eur. J.*, **2019**, *25*, 501–506, DOI: 10.1002/chem.201805073.
9. X. Zhang, I. Da Silva, R. Fazzi, A. M. Sheveleva, X. Han, B. F. Spencer, S. A. Sapchenko, F. Tuna, E. J. L. McInnes, M. Li, S. Yang and M. Schröder, Iodine Adsorption in a Redox-Active Metal−Organic Framework: Electrical Conductivity Induced by Host−Guest Charge-Transfer, *Inorg. Chem.*, **2019**, *58*, 14145–14150, DOI: 10.1021/acs.inorgchem.9b02176.
10. Z. Meng and K. A. Mirica, Two-dimensional d−π conjugated metal−organic framework based on hexahydroxytrinaphthylene, *Nano Res.*, **2021**, *14*, 369–375., DOI: 10.1007/s12274-020-2874-x.
11. Y. Jiang, I. Oh, S. H. Joo, Y. Seo, S. H. Lee, W. K. Seong, Y. J. Kim, J. Hwang, S. K. Kwak, J. Yoo and R. S. Ruo, Synthesis of a Copper 1,3,5-Triamino-2,4,6-benzenetriol Metal− Organic Framework,

J. Am. Chem. Soc., **2020**, *142*, 18346−18354, DOI: 10.1021/jacs.0c02389.

12. K. J. Erickson, F. Léonard, V. Stavila, M. E. Foster, C. D. Spataru, R. E. Jones, B. M. Foley, P. E. Hopkins, M. D. Allendorf and A. A. Talin, Thin Film Thermoelectric Metal–Organic Framework with High Seebeck Coeffi cient and Low Thermal Conductivity, *Adv. Mater.*, **2015**, *27*, 3453–3459, DOI: 10.1002/adma.201501078.
